# Impact of CRRT on graft outcomes in kidney transplantation from deceased donors with severe acute kidney injury

**DOI:** 10.1186/s12882-025-04485-6

**Published:** 2025-10-16

**Authors:** Young Ju Oh, Hyo Kee Kim, Heungman Jun, Myung-Gyu Kim, Cheol Woong Jung

**Affiliations:** 1 Department of Surgery, Korea University Anam Hospital, Korea University School of Medicine, Seoul, Republic of Korea; 2 Department of Surgery, Korea University Guro Hospital, Korea University School of Medicine, Seoul, Republic of Korea; 3 Department of Internal Medicine, Korea University Anam Hospital, Korea University School of Medicine, Seoul, Republic of Korea

**Keywords:** CRRT, AKI, Kidney transplantation, Deceased donor, Delayed graft function, Graft outcome

## Abstract

**Background:**

Kidney transplantation (KT) is the preferred treatment for end-stage renal disease. However, challenges arise when using kidneys from deceased donors (DDs) with severe acute kidney injury (AKI). This study investigated the impact of continuous renal replacement therapy (CRRT) on graft outcomes in recipients of kidneys from DDs classified as acute kidney injury network (AKIN) stage 2 or 3.

**Methods:**

A retrospective analysis was conducted on patients who received kidneys from DDs managed at Korea University Anam and Guro Hospital between 2010 and 2020. Recipients were included if the donor had AKIN stage ≥ 2 AKI. They were categorized into three groups: AKIN 2, AKIN 3 without CRRT, and AKIN 3 with CRRT. Clinical characteristics and transplant outcomes, including delayed graft function (DGF), DGF duration, number of hemodialysis (HD) sessions, biopsy-proven acute rejection (BPAR), and graft survival, were compared among groups.

**Results:**

During the study period, a total of 219 DDs were managed at our centers. Among them, 81 donors met the criteria for AKIN stage ≥ 2. From these donors, 81 recipients underwent KT, including 29 recipients who received kidneys from CRRT-treated donors. The incidence of DGF was the highest in the CRRT group (51.7%, *P* = 0.001). The CRRT group also required more HD sessions during the DGF period (2.6 ± 3.6 sessions vs. 1.6 ± 3.9 sessions, *P* = 0.001), although the CRRT group had a numerically shorter DGF duration than the non-CRRT AKIN 3 group (11.5 ± 6.9 days vs. 14.2 ± 8.7 days). The number of HD sessions was greater in the CRRT group, suggesting a more proactive perioperative support approach. BPAR rates were not significantly different across groups. One-year graft failure occurred in 5.0%, 3.1%, and 6.9% of recipients in AKIN 2, AKIN 3 non-CRRT, and AKIN 3 CRRT groups, respectively. Death-censored graft loss did not occur in any groups.

**Conclusions:**

Although CRRT was associated with a higher incidence of DGF, it might facilitate earlier recovery of graft function in recipients of kidneys from DDs with severe AKI. These findings support the use of kidneys from CRRT-treated donors, highlighting their potential to expand the donor pool without compromising one-year graft outcomes.

## Introduction

Kidney transplantation (KT) from deceased donors (DDs) with acute kidney injury (AKI) has become increasingly common due to organ shortages. While AKI has traditionally raised concerns about graft quality, recent studies suggest that kidneys from donors with AKI can provide acceptable outcomes [1–3]. These studies have primarily focused on comparisons between AKI and non-AKI donors, demonstrating that selected AKI grafts—particularly those from mild to moderate stages—can function well after KT.

However, fewer studies have evaluated outcomes from donors with severe AKI. Studies that have considered the role of renal replacement therapy (RRT), including continuous renal replacement therapy (CRRT), are even fewer. CRRT is frequently employed in critically ill donors with hemodynamic instability and severe metabolic derangements. Although its use reflects a higher severity of illness, CRRT might also stabilize the internal environment prior to procurement, potentially mitigating injury to the allograft [4].

A recent large-scale registry-based study has reported that kidneys from donors who have received dialysis are associated with a higher incidence of delayed graft function (DGF), although there is no significant difference in long-term graft or patient survival compared to non-dialysis donors [5]. Similarly, a single-center study has demonstrated that kidneys from donors requiring CRRT yield excellent one-year outcomes when carefully selected, with both graft survival and patient survival being 100% [6]. Another study has suggested that initiating CRRT after brain death in donors with oligoanuric AKI may improve hemodynamic stability and expand organ utilization, although kidney outcomes are not directly addressed in that study [4].

Despite these promising findings, prior studies have not stratified outcomes by AKI severity or explicitly evaluated the influence of CRRT as a modality distinct from other forms of RRT. Most available data treat RRT as a binary surrogate of donor severity without examining its potential therapeutic role in supporting early graft recovery [4–6].

Thus, this study aimed to address this knowledge gap by evaluating graft outcomes in recipients of kidneys from DDs with severe AKI and comparing outcomes across stratified groups based on both AKI severity and CRRT exposure.

## Methods

### Study design and patient selection

This retrospective cohort study was conducted on patients who received KT from DDs managed at Korea University Anam and Guro Hospital between 2010 and 2020. During this period, a total of 219 DDKTs were performed (197 from Anam and 22 from Guro). All of these cases represented procured kidneys that were transplanted, with no discarded cases included in the analysis. Under the Korean allocation system, one kidney is assigned to the managing center while the contralateral kidney is allocated to another institution. Therefore, among these cases, 81 adult recipients at our institution received kidneys from donors who met criteria for stage ≥ 2 AKI according to the acute kidney injury network (AKIN) classification. Donors were stratified into three groups according to AKI severity and CRRT use: AKIN stage 2 (without CRRT), AKIN stage 3 without CRRT, and AKIN stage 3 with CRRT. The CRRT group included kidneys from 29 donors (26 from Anam and 3 from Guro) who underwent CRRT before organ procurement. All transplants were performed at a single institution. Clinical data were obtained from institutional electronic medical records.

CRRT was initiated at the discretion of the attending intensivist in cases of progressive azotemia, metabolic acidosis, fluid overload, hyperkalemia, or hemodynamic instability unresponsive to conservative management. In all cases, continuous veno-venous hemodiafiltration (CVVHDF) was employed as the modality of CRRT.

The majority of CRRT sessions were initiated after the confirmation of brain death and before organ procurement; however, a minority of cases had CRRT initiated prior to the official diagnosis of brain death as part of critical care management. No standardized institutional protocol for CRRT initiation existed during the study period.

### Data collection and analysis

Baseline characteristics including age, sex, body mass index (BMI), cause of death, comorbidities (hypertension and diabetes mellitus), terminal serum creatinine (sCr), and need for CRRT were collected for both donors and recipients. For recipients, variables such as age, dialysis modality before transplantation, human leukocyte antigen (HLA) mismatch, panel reactive antibody (PRA), and presence of donor-specific antibodies (DSA) were collected. Transplant outcomes such as DGF, number of hemodialysis (HD) sessions, DGF duration, biopsy-proven acute rejection (BPAR), graft failure, and death-censored graft failure were assessed. DGF was defined as the requirement for dialysis within the first week following transplantation. DGF duration was defined as the number of days from the date of transplantation to the final dialysis session attributable to primary graft dysfunction. Graft failure was defined as irreversible loss of graft function requiring return to maintenance dialysis or re-transplantation.

### Immunosuppressive therapy and perioperative management

Induction therapy consisted of either basiliximab or rabbit anti-thymocyte globulin (rATG), as summarized in Table 2. The choice of induction agent was determined based on immunologic risk and clinical judgment. Cold ischemic time (CIT) and warm ischemic time (WIT) were also recorded for each case to assess their potential influence on early graft outcomes. Maintenance immunosuppression consisted of tacrolimus, mycophenolate mofetil/ mycophenolic acid, and corticosteroids. BPAR was treated according to standard protocols: T cell–mediated rejection (TCMR) with intravenous methylprednisolone pulse therapy, and antibody-mediated rejection (ABMR) with therapeutic plasmapheresis (plasma exchange) and intravenous immunoglobulin (IVIG) with or without rituximab.

### Statistical methods

Categorical variables were summarized as frequency and percentage, and continuous variables as mean ± standard deviation or median with interquartile range, as appropriate. Group comparisons were performed using the one-way ANOVA for normally distributed continuous variables and the Kruskal-Wallis test for non-normally distributed continuous variables. Categorical variables were compared using the Chi-square test or Fisher’s exact test, depending on distribution and sample size. All p-values were two-sided, and a p-value < 0.05 was considered statistically significant. Under the null hypothesis, we assumed no significant differences in clinical outcomes among the three donor groups stratified by AKI severity and CRRT exposure. Due to the limited sample size, only descriptive statistics and univariate analyses were conducted without multivariable modeling. All statistical analyses were performed using SPSS software version 24.0 (SPSS Inc., Chicago, IL, USA).

### Ethical considerations

The study protocol was approved by the Institutional Review Board (IRB) of Korea University Anam Hospital (IRB No. 2025-AN-0033). All procedures were conducted in accordance with the Declaration of Helsinki. The requirement for informed consent was waived by the IRB due to the retrospective nature of the study and the use of de-identified clinical data.

## Results

Baseline characteristics of donors and recipients are presented in Table 1. Donor age, history of diabetes mellitus (DM) or hypertension (HTN), and cause of death showed no significant differences among the three groups. However, donor sex showed a statistically significant difference (*P* = 0.027), with a higher proportion of male donors in the AKIN stage 3 without CRRT group (90.6%). Donor BMI was also significantly higher in the CRRT group (26.1 ± 3.9) than in the other two groups (*P* = 0.023). Additionally, last urine output before procurement was significantly lower in the CRRT group (57.8 ± 86.4 cc/day) than in AKIN 2 and AKIN 3 without CRRT groups (*P* = 0.019). KDPI scores tended to be lower in the CRRT group (57.7 ± 25.6) than in the AKIN 2 (70.4 ± 28.2) and AKIN 3 without CRRT (80.2 ± 89.5) groups, although such differences were not statistically significant (*P* = 0.091).

**Table 1:** Baseline characteristics of donors and recipients stratified by AKIN stage and CRRT use

	AKIN stage 2 (*n* = 20)	AKIN stage 3 without CRRT (*n* = 32)	AKIN stage 3 with CRRT (*n* = 29)	*P*-value
**Donors**				
Age (year)	49.1 ± 17.5	49.1 ± 11.1	46.8 ± 13.5	0.766
Male (%)	13 (65.0)	29 (90.6)	19 (65.5)	0.027
BMI	24.8 ± 2.3	23.7 ± 2.6	26.1 ± 3.9	0.023
DM (%)	4 (20.0)	8 (25.0)	6 (20.7)	0.875
HTN (%)	8 (40.0)	9 (28.1)	9 (31.0)	0.664
Cause of Death (%)				0.470
Cerebrovascular disease	11 (55.0)	19 (59.4)	14 (48.3)	
Trauma	5 (25.0)	7 (21.9)	4 (13.8)	
Hypoxic damage	4 (20.0)	6 (18.8)	11 (37.9)	
KDPI	70.4 ± 28.2	64.5 ± 25.1	57.7 ± 25.6	0.195
Last urine output (cc/day)	118.8 ± 78.2	106.2 ± 109.2	57.8 ± 86.4	0.019
**Recipients**				
Age at KT (year)	50.4 ± 11.3	53.8 ± 8.6	50.8 ± 12.4	0.384
Male (%)	10 (50.0)	24 (75.0)	32 (72.4)	0.139
HD modality immediately prior to KT (%)	15 (75.0)	23 (71.9)	23 (79.3)	0.813
BMI at pre-transplantation	23.8 ± 5.6	24.4 ± 3.5	24.9 ± 3.6	0.732
DM (%)	12 (60.0)	11 (34.3)	12 (41.1)	0.187
HTN (%)	18 (90.0)	28 (87/5)	25 (86.2)	1.000
Total HLA mismatch	4.6 ± 2.2	4.4 ± 2.0	5.2 ± 1.6	0.212
DSA (%)	1 (5.0)	1 (3.1)	1 (3.4)	1.000
PRA > 0 (%)	9 (45.0)	11 (34.3)	15 (51.7)	0.387

Abbreviations: KT, kidney transplantation; BMI, body mass index; DM, diabetes mellitus; HTN, hypertension; KDPI, Kidney Donor Profile Index; PRA, Panel Reactivity Antibody; HLA, Human Leukocyte Antigen; DSA, Donor Specific Antibodies

Recipient characteristics were largely comparable across groups. Age at transplantation, sex, BMI, dialysis modality before KT, and prevalence of DM or HTN did not differ significantly. Immunologic risk factors, including HLA mismatch, PRA > 0%, and presence of DSA, were not significantly different either among groups.

Induction therapy consisted of basiliximab or rabbit anti-thymocyte globulin (rATG), and the distribution of induction agents did not differ significantly among groups (*P* = 0.281).

CIT (241.6 ± 129.3, 290.8 ± 147.0, and 261.0 ± 136.3 min in AKIN stage 2, AKIN stage 3 without CRRT, and AKIN stage 3 with CRRT, respectively; *P* = 0.554) and WIT (30.3 ± 7.4, 29.6 ± 7.0, and 29.4 ± 8.4 min, respectively; *P* = 0.936) were also comparable among the three groups.

Graft outcomes at one year are shown in Table 2. The incidence of DGF increased significantly with AKI severity and CRRT use: 5.0% in the AKIN 2 group, 18.8% in the AKIN 3 without CRRT group, and 51.7% in the CRRT group (*P* = 0.001). Similarly, the number of HD sessions during the DGF period was significantly greater in the CRRT group (2.6 ± 3.6) than in the other two groups (*P* = 0.001).

**Fig. 1:**
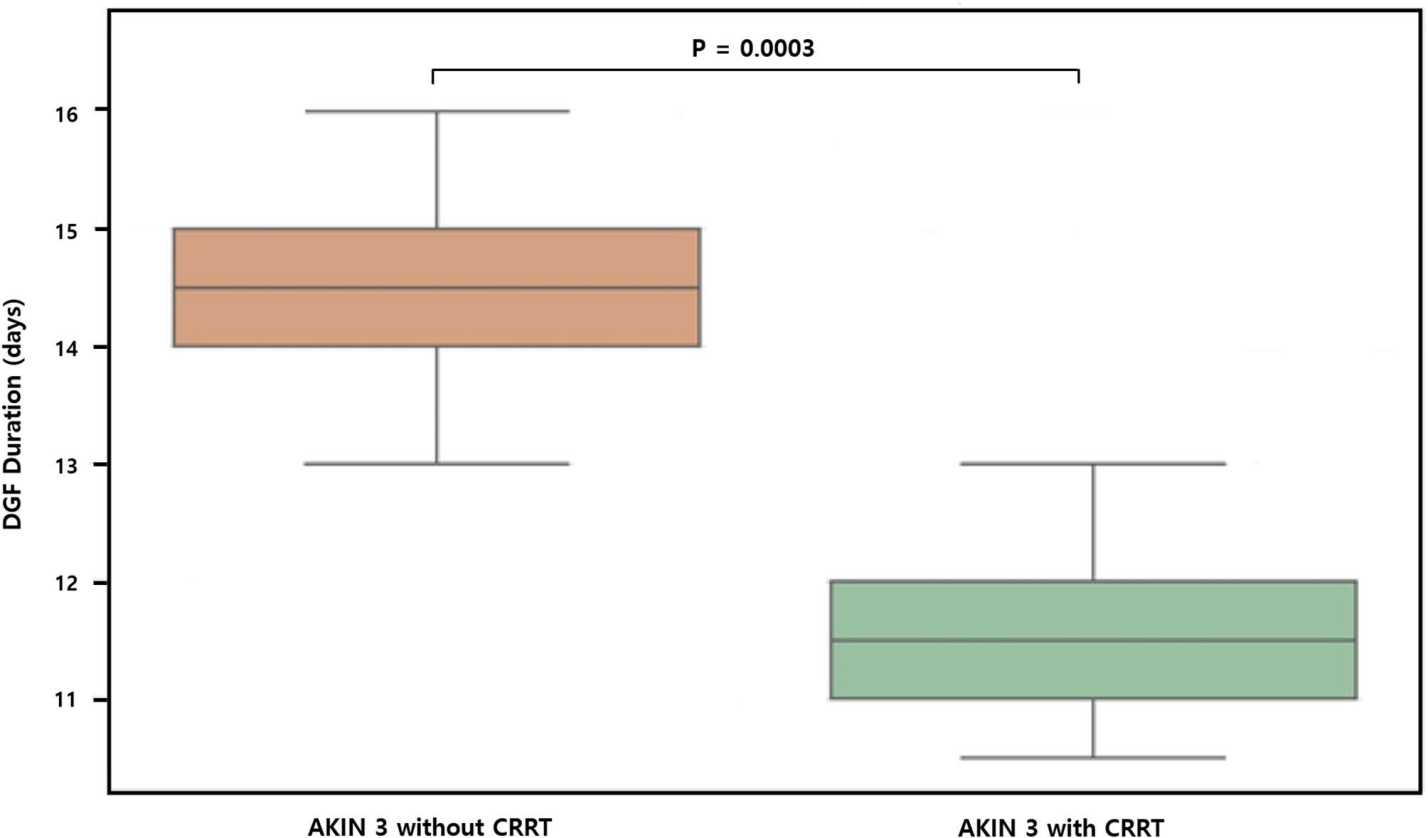
Delayed graft function duration in kidney transplant recipients from AKIN stage 3 donors with or without CRRT

Among patients who required dialysis, the average DGF duration was the longest in the AKIN 3 without CRRT group (14.2 ± 8.7 days), followed by that in the CRRT group (11.5 ± 6.9 days, *P* = 0.0003; Fig. 1). While Table 2 presents comparisons among all three donor groups, Fig. 1 was designed to focus specifically on AKIN stage 3 donors, comparing those with and without CRRT, in order to better isolate the effect of CRRT within this severity category.

**Table 2:** Graft outcomes stratified by AKIN stage and CRRT use

	AKIN stage 2 (*n* = 20)	AKIN stage 3 without CRRT (*n* = 32)	AKIN stage 3 with CRRT (*n* = 29)	*P*-value
Basilixumab induction (%)	6 (30.0)	16 (50.0)	10 (34.5)	0.281
Cold ischemic time (min)	241.6 ± 129.3	290.8 ± 147.0	261.0 ± 136.3	0.554
Warm ischemic time (min)	30.3 ± 7.4	29.6 ± 7.0	29.4 ± 8.4	0.936
DGF (%)	1 (5.0)	6 (18.8)	15 (51.7)	0.001
HD sessions during DGF	0.1 ± 0.7	1.6 ± 3.9	2.6 ± 3.6	0.001
DGF duration in patients requiring dialysis (days)	4.0 ± NA	14.2 ± 8.7	11.5 ± 6.9	
1-year graft overall graft loss (%)	1 (5.0)	1 (3.1)	2 (6.9)	0.839
Death-censored graft loss (%)	0 (100.0)	0 (100.0)	0 (100.0)	
Biopsy-proven acute rejection within 1-year (%)	3 (15.0)	3 (9.4)	7 (24.1)	0.328
Discharge Cr (mg/dL)	1.5 ± 0.6	2.0 ± 1.3	2.4 ± 1.3	0.004
1-year Cr (mg/dL)	1.4 ± 0.5	1.4 ± 0.5	1.5 ± 0.5	0.810
2-year Cr (mg/dL)	1.4 ± 0.4	1.4 ± 0.4	1.5 ± 0.5	0.858
3-year Cr (mg/dL)	1.4 ± 0.5	1.5 ± 0.5	1.4 ± 0.4	0.909
Discharge eGFR (ml/min)	48.1 ± 23.7	37.0 ± 14.2	33.6 ± 18.6	0.084
1-year eGFR (ml/min)	46.3 ± 18.5	45.9 ± 15.8	45.8 ± 18.6	0.995
2-year eGFR (ml/min)	47.4 ± 18.7	46.4 ± 15.3	46.4 ± 17.6	0.981
3-year eGFR (ml/min)	48.1 ± 19.5	46.2 ± 17.8	47.8 ± 17.9	0.926

Abbreviations: DGF, Delayed Graft Function; Cr, creatinine; eGFR; estimated glomerular filtration rate

**Fig. 2:**
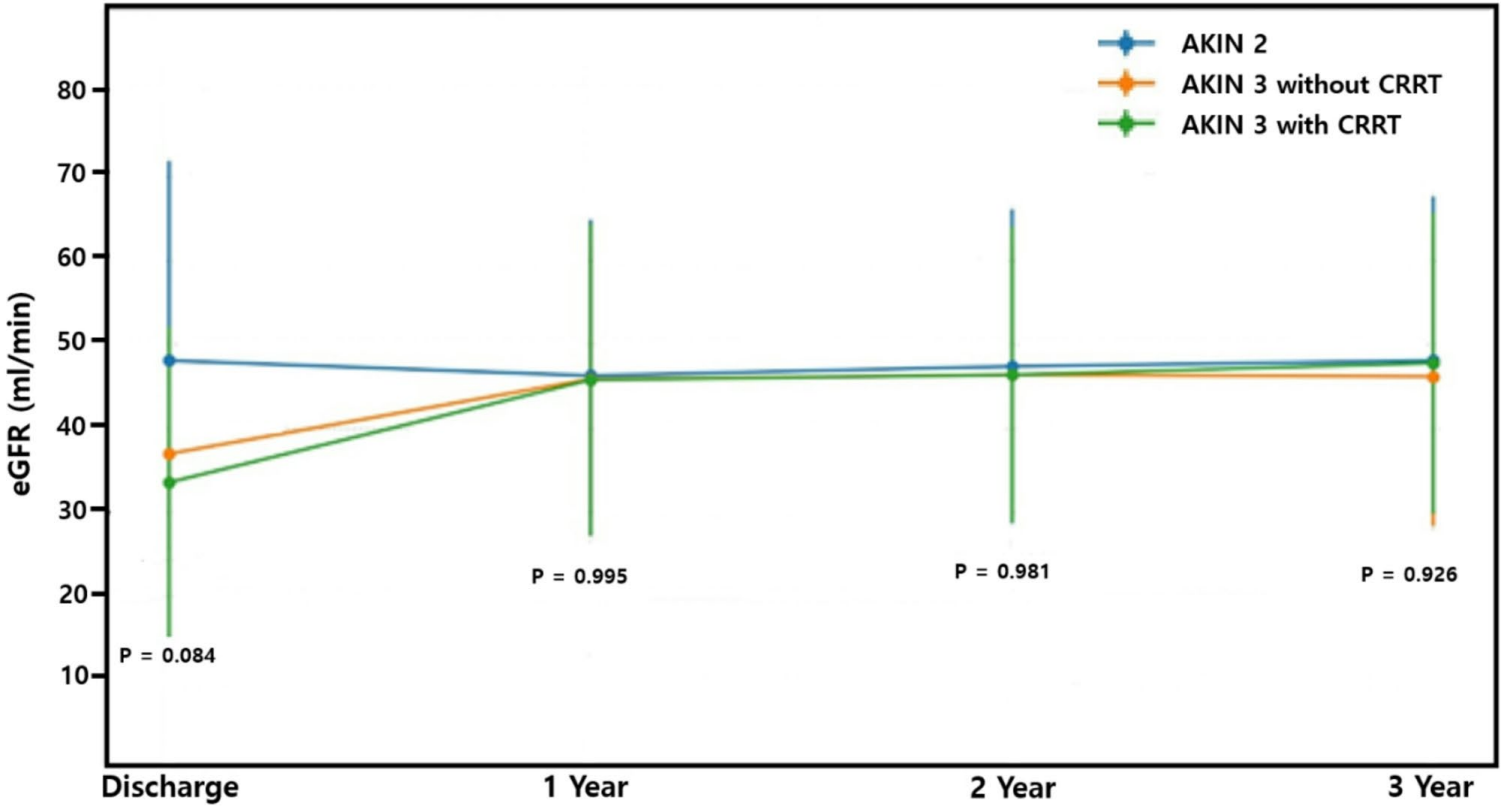
Longitudinal changes in estimated glomerular filtration rate (eGFR) from discharge to 3 years among AKIN 2 and AKIN 3 donor kidney recipients stratified by CRRT exposure

Renal function trajectories are presented in Fig. 2. Longitudinal analysis of eGFR from discharge to 3 years displayed as mean ± standard deviation revealed that recovery patterns over time were comparable across all groups, although the initial eGFR was lower in the CRRT group. No statistically significant intergroup differences were observed during follow-up.

There were no significant differences in overall graft loss at one year (*P* = 0.839). Death-censored graft failure did not occur in any groups. Rates of BPAR within the first year were not statistically different among groups (*P* = 0.328), although it was numerically higher in the CRRT group (24.1%). All rejection episodes responded to appropriate standard therapies.

## Discussion

This study evaluated the impact of CRRT on KT outcomes from DDs with severe AKI, stratified by AKI severity and CRRT exposure. Although the CRRT group showed a higher incidence of DGF, other major transplant outcomes—including graft survival, patient mortality, acute rejection, and long-term renal function—did not differ significantly among groups. These findings suggest that CRRT should not be regarded as a contraindication for KT. Instead, it may serve as a supportive intervention when used in donors carefully evaluated for reversible AKI, hemodynamic stability, absence of severe systemic infection, and favorable clinical markers after CRRT.

Findings of the present study are consistent with, and extend previous studies examining outcomes of KT using grafts from AKI donors. Prior investigations have reported that kidneys from AKI donors, including those requiring dialysis, can yield acceptable short-term and long-term results [2, 3, 7, 8]. Through a large registry-based study, Wen et al. have demonstrated that long-term graft and patient outcomes are comparable to those from non-AKI donors, although the risk of DGF is significantly higher among dialysis-requiring AKI donors [5]. Similarly, Boffa et al. have observed that although AKIN stage 3 donors are associated with higher rates of DGF and graft loss, acceptable transplant outcomes could still be achieved with appropriate donor selection [9].

In particular, favorable outcomes following transplantation of kidneys from CRRT-treated donors have been reported in several studies, with one-year graft and patient survival rates comparable to those of non-CRRT cases [6, 10]. Our study corroborated these findings, demonstrating excellent one-year graft function and survival across all groups, including those who received kidneys from donors managed with CRRT. However, it is important to note that these favorable results likely reflect careful donor selection rather than the effect of CRRT itself. Donors with potentially reversible conditions may have been preferentially selected for CRRT and subsequent organ donation.

Importantly, the present study provides insight into characteristics of DGF in this population. Although the incidence of DGF was the highest in the CRRT group, the duration of dialysis among recipients requiring it was shorter in the CRRT group than in the AKIN stage 3 non-CRRT group. This observation might reflect a more proactive or optimized perioperative management strategy in the CRRT cohort. It supports the hypothesis that CRRT may facilitate early graft recovery [4]. Furthermore, in selected donors, CRRT may help maintain hemodynamic and metabolic stability during the ICU course, which may contribute to better organ preservation and function after transplantation [4].

In addition, our longitudinal analysis of eGFR revealed that the CRRT group had significantly lower renal function at discharge, particularly when it was compared to the AKIN 2 group. However, this difference diminished over time. At 1 year and 3 years post-transplant, no statistically significant intergroup differences in eGFR were observed. These findings suggest that despite the initial severity of donor condition, recipients of kidneys from CRRT-treated donors can experience meaningful recovery of renal function over time. This is consistent with prior literature showing that even kidneys from donors with severe AKI or dialysis exposure can achieve acceptable long-term function when carefully selected [11–13].

Of note, kidney donor profile index (KDPI) scores incorporating terminal sCr and other donor-related variables were not significantly different among groups, although they tended to be lower in the CRRT cohort. This may be partly attributed to the relatively lower mean age observed in the CRRT group, suggesting a possible tendency to select donors with more favorable characteristics when deciding to utilize kidneys from CRRT-treated donors. Considering that terminal sCr is a component of KDPI calculation, CRRT can artificially reduce sCr levels, potentially leading to underestimation of donor risk [12]. Thus, lower KDPI values in the CRRT group should be interpreted with caution, as they may reflect methodological artifact rather than true donor quality.

CIT has been identified in prior studies as a major determinant of graft outcomes, with prolonged CIT increasing the risk of graft failure and mortality [14, 15]. Similarly, induction therapy can influence rejection rates, with some studies suggesting differences in outcomes between basiliximab and rATG [15]. In our cohort, both ischemic times (CIT and WIT) and induction regimen were comparable across groups, suggesting that neither ischemic injury nor induction strategy confounded the observed outcomes.

Recent literature has emphasized the importance of not excluding donor kidneys solely based on the use of renal replacement therapy. High discard rates of kidneys from AKI donors persist despite accumulating evidence supporting their safety and utility [11, 16]. Current findings contribute to this body of literature by demonstrating satisfactory graft outcomes even in the context of severe AKI with CRRT.

Moreover, dialysis duration alone does not appear to be a strong predictor of transplant success, suggesting that neither dialysis exposure nor CRRT treatment should be considered absolute exclusion criteria [12]. Previous studies have similarly shown that AKIN stage 3 donor kidneys can result in acceptable long-term outcomes [13, 17]. Further evidence supports the feasibility of utilizing such kidneys, which might otherwise be discarded based on traditional selection standards [9, 18].

Although the incidence of BPAR was not significantly different among groups, the CRRT group exhibited a numerically higher rate of rejection. This may be attributed to the higher rate and severity of DGF in this group, which has been associated with increased immunologic risk in prior studies. All rejection episodes responded to appropriate standard therapies, indicating that differences in rejection rates were unlikely to be driven by variation in immunosuppressive management but rather by donor and graft characteristics.

Given the retrospective design and relatively small sample size, multivariable analyses were not performed in this study. As a result, the potential influence of confounding factors—such as donor comorbidities, hemodynamic instability, and recipient immunologic risk—could not be fully adjusted for. Further studies with larger cohorts and multivariable modeling are warranted to better delineate the independent effect of CRRT on transplant outcomes.

This study has certain limitations. Its retrospective, single-center nature might have limited the generalizability of its findings and introduced selection bias. Although the sample size was larger than those of many prior single-institution studies, it might be insufficient to detect subtle differences in long-term outcomes. In particular, the limited sample size and low event rates (e.g., 1-year graft failure) may have reduced the statistical power to detect meaningful differences among groups. Therefore, non-significant findings should be interpreted with caution. Moreover, CRRT protocols were not standardized. In addition, unmeasured confounding factors related to donor and recipient selection might have influenced study results.

In conclusion, the present study suggests that kidneys from DDs with severe AKI requiring CRRT can be used safely in KT. Although the use of kidneys from DDs with severe AKI requiring CRRT is associated with a higher incidence of DGF and a lower initial eGFR, long-term graft function and patient survival are not compromised. Thus, CRRT might serve a supportive role in optimizing donor condition. The absence of significant differences in ischemic times and induction regimen across groups further supports that these variables did not confound the main findings. Its use should be further evaluated in prospective multicenter studies to clarify its therapeutic implications.

## Data Availability

The data generated or analyzed during this study are included in this published article.

## Abbreviations

AKI: Acute kidney injury
AKIN: Acute kidney injury Network
BPAR: Biopsy-proven acute rejection
BMI: Body mass index
CIT: Cold ischemic time
CRRT: Continuous renal replacement therapy
CVVHDF: Continuous veno-venous hemodiafiltration
DD: Deceased donor
DDKT: Deceased donor kidney transplantation
DM: Diabetes mellitus
DSA: Donor-specific antibody
DGF: Delayed graft function
eGFR: Estimated glomerular filtration rate
HD: Hemodialysis
HLA: Human leukocyte antigen
HTN: Hypertension
IRB: Institutional review board
IVIG: Intravenous immunoglobulin
KDPI: Kidney donor profile index
KT: Kidney transplantation
PRA: Panel reactive antibody
rATG: Rabbit anti-thymocyte globulin
RRT: Renal replacement therapy
sCr: Serum creatinine
TCMR: T cell–mediated rejection
WIT: Warm ischemic time
